# Intervention Effects of Okra Extract on Brain-Gut Peptides and Intestinal Microorganisms in Sleep Deprivation Rats

**DOI:** 10.1155/2022/9855411

**Published:** 2022-09-24

**Authors:** Jingru Wu, Mingxue Cao, Ming Hu, Yu Gong, Jianming Xue, Yilin Yang, Hairui Zhou

**Affiliations:** ^1^Department of Pharmacy, The Second Affiliated Hospital of Jiamusi University, No. 522, Hongqi Street, Jiamusi 154004, Heilongjiang, China; ^2^Department of Pharmacy, Peking University People's Hospital, No. 11, Xizhimen South Street, Beijing 100044, China; ^3^Department of General Surgery, The First Affiliated Hospital of Jiamusi University, No. 348, Dexiang Street, Jiamusi 154003, Heilongjiang, China; ^4^Deparment of Instrument, The First Affiliated Hospital of Jiamusi University, No. 348, Dexiang Street, Jiamusi 154003, Heilongjiang, China; ^5^College of Pharmacy, Jiamusi University, No. 148, Xuefu Street, Jiamusi 154007, Heilongjiang, China; ^6^Deparment of Medicinal Chemistry, College of Pharmacy, Jiamusi University, No. 148, Xuefu Street, Jiamusi 154007, Heilongjiang, China

## Abstract

**Objective:**

Okra, possessing various bioactive components, is used to treat different diseases. This study sought to estimate the intervention effects of okra extract (OE) on brain-gut peptides (BGPs) and intestinal microorganisms in sleep deprivation (SD) rats.

**Methods:**

SD rat models were established using the modified multiple platform method and then treated with normal saline, diazepam tablets, or different doses of OE. Body weight and average daily water consumption of rats were recorded. Depressive behaviors of rats were assessed by the open field test and sucrose preference test. Serum levels of noradrenaline, melatonin, inflammatory factors (IL-1*β*/IL-6/TNF-*α*/IL-4/IL-10), and BGP indexes, including gastrin (GAS), motilin (MTL), 5-hydroxytryptamine (5-HT), cholecystokinin (CCK), and vasoactive intestinal peptide (VIP) were measured by ELISA. Additionally, the DNA relative contents of representative intestinal microorganisms in the collected rat feces were determined using RT-qPCR.

**Results:**

SD decreased body weight and average daily water consumption and induced depressive behaviors as well as stress and inflammatory responses in rats. SD rats exhibited lowered GAS, MTL, 5-HT, and VIP but elevated CCK and showed diminished DNA relative contents of *Bacteroidetes* and probiotics (*Bifidobacteria* and *Lactobacilli*) but increased *Clostridium perfringens*. OE at different doses ameliorated the depressive behaviors and mitigated the stress and inflammatory responses in SD rats, raised the serum contents of GAS, MTL, 5-HT, and VIP, reduced CCK level, elevated the DNA relative contents of *Bacteroidetes* and probiotics, but diminished *Clostridium perfringens*. OE exhibited similar intervention effects to diazepam tablets (positive control).

**Conclusion:**

OE exerts intervention effects on BGPs and intestinal microorganisms in SD rats.

## 1. Introduction

Sleep is ubiquitous and complicated animal behavior, and sleep health is a multidimensional pattern of sleep-wake, adapted to environmental, individual, and social demands, facilitating physical and mental well-being [1, 2]. Sleep deprivation (SD) is a result of multiple health problems or a contributor to various major health risks, raising great public health concerns [3]. SD occurs when insufficient sleep results in deterioration in health, decreased performance, and inadequate alertness, which is extremely common, with approximately 20% of adults being sleep-deprived [4]. In addition, SD contributes to changes in mood, memory, learning, and immune functions, affecting physical or psychological activities [5] and causing energy metabolism disorder in neural cells [6]. As a strong stress stimulus, prolonged SD impacts the behavioral and physiological functions of the body, increases general systemic diseases, and leads to cognitive decline and chronic fatigue syndrome [7]. Owning to the importance of sleep, it is necessitated to underline the complicated mechanism of SD to enhance sleep quality.

Brain-gut peptides (BGPs), such as motilin, gastrin, and calcitonin gene-related protein [8], are dual-distributed in the gastrointestinal and nervous systems, exhibiting roles in regulating the secretion of pro-inflammatory cytokines [9]. More importantly, the neuroprotective effects of numerous BGPs have been documented [10]. For instance, electroacupuncture treatment could palliate the neurodegenerative disease—Parkinson's disease—by regulating BGPs in rats, including cholecystokinin, gastrin, neuropeptide *Y*, somatostatin, and peptide YY [11]. Furthermore, BGPs are implicated in sleep, learning from the idea that ghrelin is a BGP that causes anxiety and other aberrant emotions, contributing to the impacts of insomnia on emotional behaviors [12].

Intestinal microorganism, involved in various physiological processes and metabolism, is imperative in circadian rhythms and sleep [13]. Interestingly, the brain is intimately and complexly linked with the gut, and this bidirectional communication between the central nervous system and the intestinal nervous system is termed the brain-gut axis, in which the gut microbiome is pivotal [14]. Prior work has supported the correlation between intestinal flora and sleep quality [15, 16], and SD can result in corticosterone increase, intestinal flora disorder, and colitis phenotype in mice [17, 18]. Transient SD contributes to subtle changes in intestinal microorganisms in mice [19], whereas intestinal microorganisms modulate SD-elicited inflammatory responses and cognitive impairment [20]. There are more than 100 trillion microorganisms in the human gut and *Bacteroidetes* are reported to be a common type of intestinal bacteria [21], which are changed in SD mice [22]. In the wake of advances in clinical medicine, the role of intestinal flora in the brain-gut axis is gradually recognized [23, 24], which has emerged as a research hotspot. It is noteworthy that the brain-gut-microbiome axis imposes a potential role in Alzheimer's disease [23]. Neuropeptide *Y* and peptide YY exert vital roles in obesity development through the brain-gut axis [25]. Imbalances in the structure and function of intestinal flora may be a driving force in global inflammation, which is implicated in several diseases [26, 27]. However, more investigation regarding the effect of SD on BGP and intestinal microorganisms remains to be carried out.

Okra (*Abelmoschus esculentus* L. Moench), originated in Africa, is a vital vegetable crop that grows widely in tropical, subtropical, and warm temperate regions [28, 29], which also belongs to the natural plant-derived drug [30]. In 2016, the production of okra exceeded 8.9 million tons in more than 33 countries, with the highest production in India [31]. Okra fruit, used as a nutritious and fresh vegetable, possesses medicinal and health properties. Additionally, okra possesses abundant functional components, such as lipids, minerals, carbohydrates, dietary fiber, pectin, amino acids, and vitamins [32]. Okra extract (OE) mainly consists of okra fruit extract and okra seed extract, playing a myriad of effects. There is evidence to suggest that okra fruit powder can affect metabolic syndrome and intestinal microflora diversity in high-fat-diet-induced obese mice [33]. In particular, okra seed oil palliates ethanol-elicited liver damage and mediates intestinal microbial dysregulation in mice [34]. Okra polysaccharide exerts an antidepressant effect by balancing gut microbiota [35]. Okra seed and leaf extracts exert a significant antioxidant activity and can act as major antioxidants [36]. The ethanol extract of okra fruit may be an effective wound-healing agent with antibacterial, antioxidant, and anti-inflammatory activities [37]. Total polyphenols, the major components in okra fruit extract [38], have several subclasses, such as flavonoid. Flavonoid compounds are the most imperative active ingredients in okra [39, 40]. Previous studies have also reported the isolation of natural free flavonoid compounds and their derivatives from okra fruit [41–43]. It is noteworthy that flavonoid compounds are capable of regulating intestinal bacteria and playing a defensive role against diseases [44–47]. However, there are few clinical and experimental studies at home and abroad to assess the effects of okra fruit extract on SD-induced intestinal flora imbalance and BGPs. Hence, in this study, Sprague-Dawley rats were used as the experimental animals to establish SD rat models by the modified multiple platform method and SD rats were intragastrically administered with different doses of OE to explore the intervention effect of OE on BGPs and intestinal microorganisms in SD rats, hoping to provide a scientific theoretical basis for OE in maintaining human intestinal microbial homeostasis and improving public health issues such as SD in humans. In addition, this study lent insight to develop a natural plant-derived drug rich in flavonoid compounds, along with the potential to adjust intestinal bacteria, so as to improve the SD-caused body behaviors.

## 2. Materials and Methods

### 2.1. Ethics Statement

All animal experiments were conducted in line with the *Guide for the Care and Use of Laboratory Animals* and ratified by the Laboratory Animal Ethics Review Committee of Jiamusi University. Enormous efforts were made to minimize the animal quantity and their pain.

### 2.2. Experimental Animals

Healthy male Sprague-Dawley rats (aged 15 weeks and weighing 200 ± 20 g) purchased from Charles River [License No. SCXK (Beijing) 2016-0008, Beijing, China] were reared at 18–25°C and 40–60% humidity on 12 h light-dark cycles, with adequate food and water [5, 8], subjecting to adaptive feeding for one week.

### 2.3. Establishment of SD Models

A modified multiple platform method was used to establish rat models of SD [5]. Four small cylindrical platforms (6.5 cm diameter and 8 cm height) were fixed at the bottom of a self-made SD box (41 cm × 33 cm × 25 cm) with an interplatform spacing of 8 cm longitudinally and 12 cm transversely. The box was filled with water and the water surface was approximately 1.0 cm below the platform surface. The rats were placed on platforms where they had free access to moving, food, and water, with ample water and food on the top of the platforms. Additionally, the top of the box was covered with wire netting to prevent rats from escaping. The illumination of the 40-w fluorescent lamp was given from 6 : 50 to 18 : 50. The water temperature was maintained at 23–25°C, with water in the box changed every day to keep clean. When entering the rapid eye movement (REM) phase of sleep, the rat head would fall into the water due to muscle relaxation and reduced whole-body muscle tension, and then, the rats would wake up and try to climb up the platform to avoid drowning [22].

During the adaptive feeding, except the control group, rats in other groups received standing training in the SD box once a day for 1 h each. SD was processed starting on the 8th day, once a day for 12 h (6 : 50–18 : 50) each, lasting for 3 weeks. The general states of rats, such as mental status, circadian activity, coat color, and stress response, were observed, and the daily water consumption was recorded.

### 2.4. Drug Administration and Grouping

Totally 48 rats were randomly allocated into the following 6 groups (8 rats/group, rats were intragastrically injected with different drugs): control group (normal saline, 8 mL/kg/d), SD group (normal saline, 8 mL/kg/d), and positive control group (diazepam tablet suspension); and low-dose OE group (L-OE, 5 mg/kg/d), medium-dose OE group (M-OE, 10 mg/kg/d), and high-dose OE group (H-OE, 20 mg/kg/d) [33]. During the SD process, diazepam tablets and OEs were dissolved in normal saline to make into suspensions, which were intragastrically administrated into rats daily. The diazepam tablets were provided by Yimin Pharmaceutical (H11020898, Beijing, China), and the dose for rats was calculated to be 0.45 mg/kg/d based on 6.3 times of adult dosage. OE was provided by Biocar Biotechnology (Guangzhou, Guangdong, China), showed as brown fine powders.

After 3 weeks of SD, the open field test (OFT) and sucrose preference test (SPT) were conducted. The sample collection was performed the next morning after all behavioral experiments. Following anesthesia, blood was collected from the abdominal aorta of rats using a 10-mL vacuum blood collection tube (at least 5 mL per rat) and then allowed to stand for 30 min, followed by centrifugation at 3000 × *g* for 15 min to obtain the serum [33, 34]. In addition, rats were stimulated by tail lifting to promote defecation on the ultra-clean bench, with at least one grain of formed feces obtained from each rat. Each grain of feces was stored in a sterile cryopreservation tube, and the obtained serum and feces were placed in the −80°C freezer for subsequent analyses.

### 2.5. Determination of Body Weight

Rats were weighed using an electronic scale (Yajin Electronic Technology, Shanghai, China) on days 0, 7, 14, and 21 of the SD process, with the unit of g.

### 2.6. Open Field Test (OFT)

The OFT analysis was used for behavioral examination in rats. After 3 weeks of SD, rats were placed in an open box (100 cm × 100 cm × 40 cm). The bottom of the box was segmented into 25 equally sized grids (20 cm × 20 cm) with black lines, and the grids along the side walls were calculated as peripheral grids (16 pieces) and the rest were central grids (9 pieces). Subsequently, we manually counted the number of rats horizontally crossing the grids within 5 min (the scores of horizontal movements: 1 score, all four paws entered the grid, counted as 1 score/grid) and the number of rearing of hind limbs (the scores of vertical movement: 1 score, both forepaws rose high into the air or climbed the wall, counted as 1 score/time). The OFT scores were the sum of the above two. This test was conducted by two experimenters who did not know the grouping of experimental rats in advance. The scores recorded by the two experimenters were averaged to obtain the final score. Following the test for each rat, the open box was cleaned with 75% ethanol and thoroughly dried, after which the next rat was observed to avoid odors affecting the test results.

### 2.7. Sucrose Preference Test (SPT)

After 3 weeks of SD, rats were subjected to the SPT. Prior to the test, rats fasted and water was forbidden for 24 h. Subsequently, rats were given two bottles of water quantified in advance: one bottle of 1% sucrose water and the other bottle of pure water. Thirty min later, the positions of sucrose water and pure water were changed. Sixty min later, the water bottle was taken away and the weight was recorded. The proportion of sucrose water consumption (sucrose preference rate) = sucrose water consumption/total water consumption × 100%.

### 2.8. Enzyme-Linked Immunosorbent Assay (ELISA)

ELISA kits (Enzyme-linked Biotechnology, Shanghai, China) were used to determine the levels of noradrenaline (NE, ml002873), melatonin (MT, ml003417), interleukin (IL)-1*β* (ml037361), IL-6 (ml064292), tumor necrosis factor-*α* (TNF-*α*, ml002859), IL-4 (ml058093), IL-10 (ml028497) [22], and BGP-related indexes, including gastrin (GAS, ml862256), motilin (MTL, ml003079), cholecystokinin (CCK, ml003073), 5-hydroxytryptamine (5-HT, ml059511), and vasoactive intestinal peptide (VIP, ml936589) [8, 24].

### 2.9. Detection of Representative Intestinal Microorganisms

Fecal samples (0.2 g) were put into a 2-mL round-bottom centrifuge tube, and the total DNA was extracted from feces using fecal DNA extraction kits. The *V*3–*V*4 variable region sequences of the 16S rRNA gene in the four investigated intestinal bacteria (*Bifidobacteria*, *Lactobacilli*, *Clostridium perfringens*, and *Bacteroidetes*) acted as the targets. The primers were designed based on the conserved region in the sequence, and then, the amplification of polymerase chain reaction was processed as follows: 5 min at 95°C, then 25 cycles of 30 s at 95°C, 30 s at 50°C, 40 s at 72°C, and extension for 7 min at 72°C. Data were analyzed using the 2^−ΔΔCt^ method, and the primers are exhibited in Table 1.

### 2.10. Statistical Analysis

All data were processed using SPSS 21.0 software (IBM Corp. Armonk, NY, USA). Measurement data were exhibited as mean ± standard deviation. Data between two groups were analyzed by the independent sample *t*-test or one-way repeated measures analysis of variance (ANOVA). Comparisons between multiple groups were conducted using one-way ANOVA, and Tukey's multiple comparisons test was performed for the post hoc analysis. The *p* < 0.05 was regarded statistically significant.

## 3. Results

### 3.1. SD-Induced Depressive Behaviors in Rats

**Figure 1:**
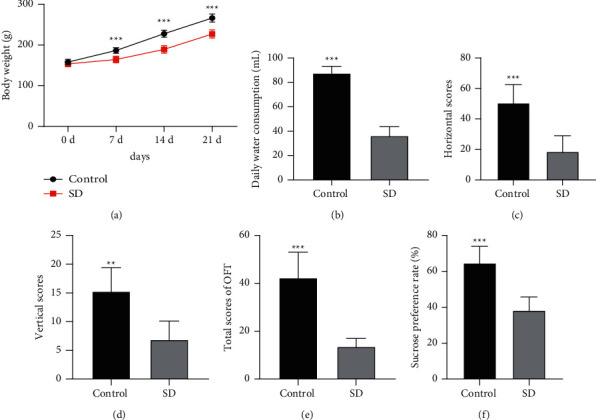
SD-induced depressive behaviors in rats. SD rat models were established using a modified multiple platform method. (a) Assessment of body weight; (b) statistics of average daily water consumption; (c)–(e) assessment of OFT scores; (f) determination of sucrose preference rate. *N* = 8, data were expressed as mean ± standard deviation. Data comparisons in panel *A* were performed using one-way repeated measures ANOVA and data in panels *B*/*C*/*D*/*E*/*F* were analyzed using the independent sample *t*-test. ^*∗∗*^*p* < 0.01, ^*∗∗∗*^*p* < 0.001.

Growing reports have elicited the association between SD with depression- and anxiety-like behaviors [48, 49]. To assess the effect of SD on depressive behaviors in rats, we observed the general states of rats after SD modeling and then found that SD rats showed manifestations of excitement, irritability, listlessness, and reduced response to surrounding environmental stimuli, with irregular and dull coat color. Compared with the control group, the SD rats had significantly reduced body weight on days 7, 14, and 21 (*p* < 0.01) (Figure 1(a)) and diminished average daily water consumption (*p* < 0.01) (Figure 1(b)). In addition, the OFT results revealed that SD rats exhibited lower horizontal scores, vertical scores, and total scores than the control rats (*p* < 0.01) (Figures 1(c)–1(e)). The SPT results unveiled that relative to the control group, SD rats had notably diminished sucrose preference rate (*p* < 0.01) (Figure 1(f)). Overall, SD could cause depressive behaviors in rats.

### 3.2. SD-Triggered Stress and Inflammatory Responses in Rats

**Figure 2:**
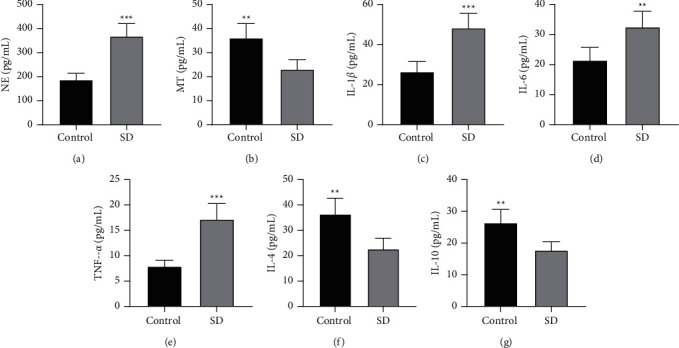
Effects of SD on stress and inflammatory responses in rats. (a)-(b) ELISA determined the serum levels of NE and MT; (c)–(g) ELISA measured the serum levels of inflammatory factors (IL-1*β*, IL-6, TNF-*α*, IL-4, and IL-10). *N* = 8, data were presented as mean ± standard deviation, and the independent sample *t*-test was used for comparisons between groups. ^*∗∗*^*p* < 0.01, ^*∗∗∗*^*p* < 0.001.

It is noteworthy that SD affects the balance of inflammatory markers and cytokines [50, 51] and induces stress responses in mice [22, 52]. Next, we measured the serum levels of NE, MT, and inflammatory factors in SD and control rats by ELISA, and observed that SD rats presented prominently higher NE but lower MT than the control group (all *p* < 0.01) (Figures 2(a) and 2(b)). Additionally, compared with the control group, the levels of pro-inflammatory factors (IL-1*β*, IL-6, and TNF-*α*) were markedly elevated (*p* < 0.01) (Figures 2(c)–2(e)), while the levels of anti-inflammatory cytokines (IL-4 and IL-10) were significantly diminished in the SD group (*p* < 0.01) (Figures 2(f) and 2(g)). In summary, SD could result in stress and inflammatory responses in rats.

### 3.3. OE Improved Depressive Behaviors and Mitigated Stress and Inflammatory Responses in SD Rats

**Figure 3:**
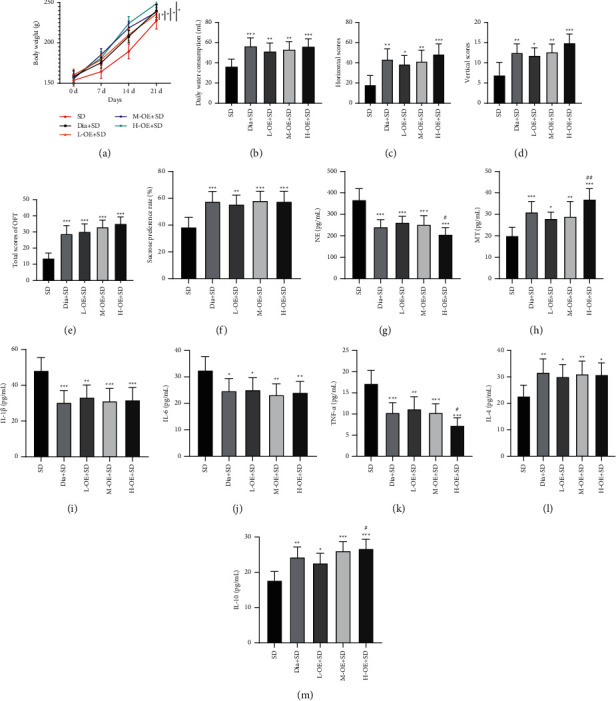
OE improved depressive behaviors and mitigated stress and inflammatory responses in SD rats. Rats were subjected to SD along with intragastric administration. (a) Assessment of body weight; (b) statistics of average daily water consumption; (c)–(e) assessment of OFT scores; (f) determination of sucrose preference rate; (g)-(h) measurement of NE and MT levels in serum; (i)–(m) ELISA determined the levels of inflammatory factors (IL-1*β*, IL-6, TNF-*α*, IL-4, and IL-10). *N* = 8, data were expressed as mean ± standard deviation. One-way ANOVA was used for comparisons among multiple groups, followed by Tukey's test. Compared with the SD group, ^*∗*^*p* < 0.05, ^*∗∗*^*p* < 0.01, ^*∗∗∗*^*p* < 0.001; compared with the L-OE + SD group, ^#^*p* < 0.05, ^##^*p* < 0.01.

Preceding evidence has noted that okra polysaccharide produces antidepressant effects by preventing inflammation and balancing intestinal microflora [35]. To investigate the effects of OE on depressive behaviors, stress, and inflammatory responses in SD rats, we intragastrically administrated 5, 10, and 20 mg/kg/d OE into rats during the SD process. The results revealed that relative to the SD group, OE intervention noticeably maintained the elevation in rat body weight (*p* < 0.01), average daily water consumption, OFT scores, and sucrose preference rate (*p* < 0.01), while no differences were evident between the low-, medium-, and high-dose groups (all *p* > 0.05) and between the OE group and the diazepam group (all *p* > 0.05) (Figures 3(a)–3(f)). Furthermore, after OE intervention, the NE level was significantly diminished, while the MT level was remarkably raised in SD rats, with statistically evident differences in NE and MT levels between the L-OE + SD group and the H-OE + SD group (all *p* < 0.01), but not between the L-OE + SD group and the M-OE + SD group (*p* > 0.05) (Figures 3(g) and 3(h)). Finally, we determined the inflammatory factor levels in rat serum and noted that OE attenuated the stimulatory effect of SD on serum cytokines, manifested by significantly reduced IL-1*β*, IL-6, and TNF-*α*, but there were no obvious differences in IL-1*β* and IL-6 between the M-OE/H-OE + SD groups and L-OE + SD group (all *p* > 0.05) and the difference in TNF-*α* level between the L-OE + SD group and H-OE + SD group was statistically evident (*p* < 0.01) (Figures 3(i)–3(k)). Additionally, in comparison with the SD group, OE intervention clearly enhanced IL-4 and IL-10 (all *p* < 0.01) and no differences were significant in IL-4 level between the low-, medium-, and high-dose groups (*p* > 0.05), and IL-10 level was markedly higher in the H-OE + SD group than that in the L-OE + SD group (*p* < 0.01), and the OE group and the diazepam group showed no obvious differences (*p* > 0.05) (Figures 3(l) and 3(m)). Briefly, OE could ameliorate depressive behaviors and alleviate stress and inflammatory responses in SD rats.

### 3.4. OE Regulated the Secretion of BGPs in SD Rats

**Figure 4:**
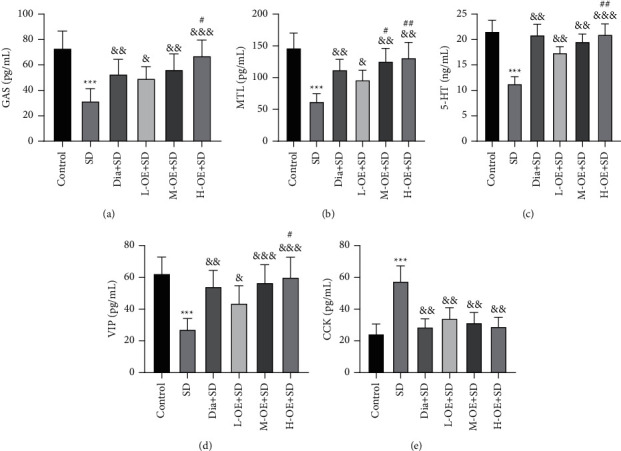
Regulatory effect of OE on BGP secretion in SD rats. (a)–(e) GAS, MTL, 5-HT, VIP, and CCK levels measured by ELISA kits. *N* = 8, data were exhibited as mean ± standard deviation. One-way ANOVA was adopted for comparisons among multiple groups, followed by Tukey's test. SD group compared with the control group, ^*∗∗∗*^*p* < 0.001; OE groups compared with the SD group, ^&^*p* < 0.05, ^&&^*p* < 0.01, ^&&&^*p* < 0.001; compared with the L-OE + SD group, ^#^*p* < 0.05, ^##^*p* < 0.01.

The insomnia-triggered anxiety behaviors have been identified to be related to changes in BGPs [12]. To assess the function of OE on BGPs in SD rats, we measured the serum levels of BGP-related indexes (GAS, MTL, 5-HT, VIP, and CCK) in rats using ELISA and noted that the SD group exhibited lower GAS, MTL, 5-HT, and VIP and higher CCK than the control group. However, OE intervention reversed the SD-induced changes in the above indexes. Meanwhile, we observed that the H-OE + SD group had higher GAS, 5-HT, and VIP than the L-OE + SD group, and M-OE/H-OE + SD groups exhibited a higher MTL level than the L-OE + SD group (all *p* < 0.01), but there was no evident difference in the CCK level among different dose groups and between the OE group and diazepam group (all *p* > 0.05) (Figures 4(a)–4(e)). Taken together, OE could modulate the BGP generation in SD rats.

### 3.5. Intervention Effects of OE on Representative Intestinal Microorganisms in SD Rats

**Figure 5:**
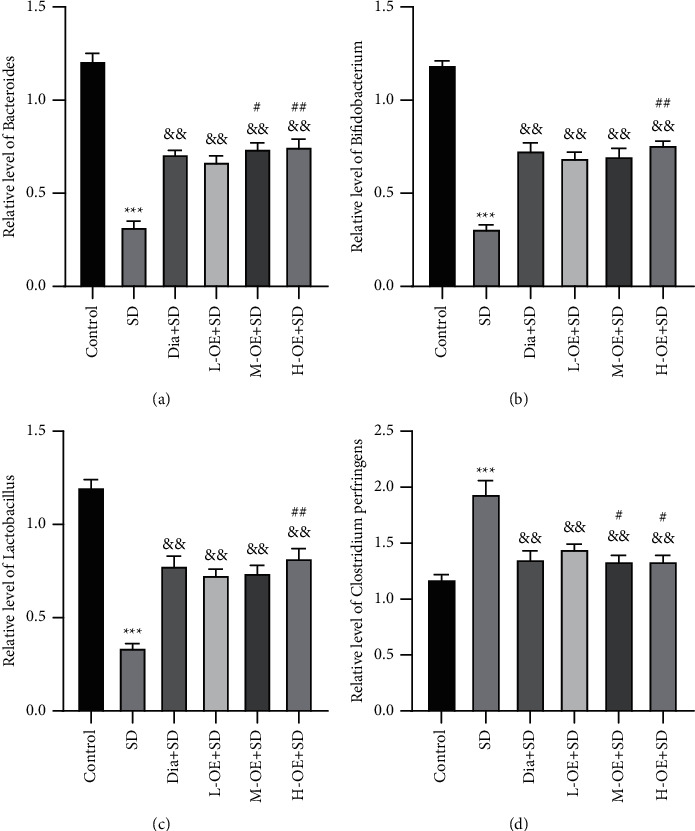
Intervention effects of OE on intestinal microorganisms in SD rats. (a)–(d) DNA relative contents of *Bacteroidetes*, *Bifidobacteria*, *Lactobacilli*, and *Clostridium perfringens* detected by RT-qPCR. *N* = 8, data were expressed as mean ± standard deviation. Comparisons between groups were conducted using one-way ANOVA, followed by Tukey's test. The SD group compared with the control group, ^*∗∗∗*^*p* < 0.001; OE groups compared with the SD group, ^&&^*p* < 0.01; compared with the L-OE + SD group, ^#^*p* < 0.05, ^##^*p* < 0.01.

Owing to the significance of intestinal microorganisms in physiological rhythm and sleep [13], we determined the DNA relative contents of four representative intestinal bacteria by RT-qPCR. Subsequently, we observed that compared with the control group, the DNA relative content of *Bacteroidetes* was clearly diminished in the SD group but raised after OE intervention, with significant differences between medium-/high-dose OE and low-dose OE (all *p* < 0.01) and no significant differences between the diazepam group and the OE group (*p* > 0.05) (Figure 5(a)).

Moreover, the SD group had noticeably lower DNA relative contents of probiotics (*Bifidobacteria* and *Lactobacilli*) than the control group (*p* < 0.01), indicating the obvious inhibition of SD on these two intestinal bacteria. After OE intervention, the DNA relative contents of *Bifidobacteria* and *Lactobacilli* increased significantly (*p* < 0.01), and this increase was greater in the H-OE + SD group than that in the L-OE + SD group (Figures 5(b) and 5(c)).

In addition, the DNA relative content of *Clostridium perfringens* was elevated in SD rats but reduced upon OE intervention, which was markedly lower in the *M*-OE/H-OE + SD groups than that in the L-OE + SD group (all *p* < 0.01), with no evident difference between the diazepam group and the OE group (*p* > 0.05) (Figure 5(b)). Altogether, OE exerted an intervention effect on representative intestinal microorganisms in SD rats.

## 4. Discussion

Sleep is essential for physiological homeostasis maintenance, health, and life quality, but SD is a growing health issue in contemporary society [53, 54]. Recently, the relevant research about SD mainly focuses on the cognitive function of the brain, and for instance, SD is reported to disrupt the plasticity and transmission of synapses, resulting in memory impairment [55]. Propofol alleviates learning and memory impairment in SD rats by suppressing autophagy in hippocampal neurons [6]. Chronic SD causes memory impairment, and selenium treatment improves antioxidant enzyme activity in the hippocampus [56]. Compelling evidence has identified that the gut microbiome influences sleep quality via the brain-gut-microbiome axis [15]. Importantly, the components of okra, such as polysaccharides and pectin, could regulate intestinal flora [57]. The current study highlighted the intervention effects of OE on BGPs and intestinal microorganisms in SD rats.

SD is reported to have numerous negative effects, including cognitive deficits, impaired performance, personal conflicts, mood disturbances, and poor communication with family [58]. A preceding meta-analysis has suggested that SD can exacerbate depression-like behaviors [59]. A core symptom of depression-anhedonia that refers to the inability to experience pleasure from enjoyable or rewarding activities can be evaluated by the SPT assay, and the OFT experiment is a test of anxiety-related behaviors and locomotion [60]. Herein, we estimated the association between SD and depressive behaviors. Our results noted that SD rats exhibited decreased body weight, daily water consumption, OFT scores, and sucrose preference rate. In line with our findings, mice subjected to prolonged paradoxical SD exhibited depressive-like behaviors and decreased locomotive activity and sucrose preference rate [61]. Additionally, SD rats exhibited anxiety-like behavior during OFT and depression-like behaviors in the forced swim test [62]. Intriguingly, 96 h of REM-SD enhanced caloric intake but reduced body weight in rats [63]. Conjointly, SD could provoke depressive behaviors in rats.

Growing reports have revealed that SD could increase oxidative stress and inflammatory markers [64, 65]. Stress normally results in sleep loss which, in turn, could aggravate physiological stress responses [66]. MT is an acknowledged free radical scavenger with powerful antioxidant and antistress functions [67, 68]. NE is involved in stress responses and stressful stimuli could induce an increased NE level in the prefrontal cortex [69]. Due to the intrinsic impacts of SD on inflammation and stress response, we detected the relevant indexes. Unsurprisingly, SD rats had elevated NE; diminished MT; raised IL-1*β*, IL-6, and TNF-*α*; and reduced IL-4 and IL-10. Consistently, previous findings supported that SD exacerbates inflammatory responses, featured by an elevation of pro-inflammatory mediators including IL-1*β*, IL-6, IL-17, and TNF-*α* in humans and animals [22, 70, 71]. Briefly, the above evidence and findings confirmed that SD caused stress and inflammatory responses in rats.

Apart from the nutritious feature, okra possesses antioxidant, immunomodulation, and antifatigue effects, exerting health-promoting properties [72]. To assess the role of OE in SD rats, we intragastrically injected different doses of OE into SD rats. Afterwards, we observed that OE intervention enhanced body weight, daily water consumption, OFT scores, and sucrose preference rate in SD rats. Additionally, OE treatment lowered NE; increased MT; diminished IL-1*β*, IL-6, and TNF-*α*; and elevated IL-4 and IL-10 in SD rats. Importantly, accumulating studies have evidenced the antidepressant effects of numerous herbs [73–75]. OE, abundant in antioxidant substances, suppresses oxidative stress by avoiding the excessive consumption of antioxidant enzymes [76]. Moreover, okra is a truly promising natural ingredient to reduce inflammation in individuals with inflammation-predisposed disorders, with the potential to diminish C-reactive protein, IL-1*β*, IL-6, and TNF-*α* [77]. In particular, flavonoid, one of the most pivotal components of okra, is responsible for the antioxidant and anti-inflammatory effects [39]. Compelling evidence suggests that the total flavonoid extract of flowers of *Abelmoschus manihot* (L.) Medicus alleviates acute lung injury in mice by inhibiting inflammation and oxidative stress [78]. In addition, total flavonoids extracted from flowers of *Abelmoschus manihot* can protect mice against carbon tetrachloride-induced liver injury via antioxidant stress and anti-inflammatory properties [79]. Collectively, OE intervention mitigated the SD-evoked depressive behaviors and stress and inflammatory responses in rats.

The possible involvement of brain-gut interactions in insomnia treatment has been confirmed [80]. Subsequently, we determined the BGP-related indicators and unraveled that GAS, MTL, 5-HT, and VIP levels were decreased, and the CCK level was increased in SD rats, while these SD-caused changes were annulled by OE intervention. GAS is a brain-gut peptide that is widely known for its growth hormone-releasing and orexigenic activities, which participates in sleep regulation [81]. Central 5-HT limits apnea and stabilizes breathing by reducing cholinergic signaling through muscarinic receptors [82]. However, the studies about the function of OE in regulating BGPs are limited, and our findings creatively documented that OE could regulate the BGP secretion in SD rats.

Sleep duration and quality may be essential targets for keeping healthy gut microbiota composition [16], and sleep disorders are also linked with changes in gut microbiota composition in animal and adult human models [83]. *Lactobacilli* and *Bifidobacteria* are beneficial bacteria in the gut [15, 84, 85], and *Lactobacillus fermentum* can improve sleep conditions in mice and effectively reduce sleep latency and the time to return to normal REM sleep [86]. Moreover, the combination of living *Bifidobacterium* and *Lactobacillus* mitigates anxiety- and depression-like behaviors in mice [87]. On a separate note, *Clostridium perfringens* is a class of harmful bacteria present in the gut [88], which can lead to intestinal dysbacteriosis [89]. We then detected the DNA relative contents of four intestinal bacteria. The RT-qPCR results unveiled that SD rats showed elevated DNA relative contents of *Clostridium perfringens* and diminished *Bacteroidetes*, *Bifidobacteria*, and *Lactobacilli*. By contrast, OE reversed the above SD-elicited changes in intestinal bacteria. Preceding evidence has noted that okra pectin palliates inflammatory responses and protects the intestinal mucosal barrier function by increasing the content of intestinal antibacterial peptides [57]. Moreover, okra seed oil notably enhances the *Bacteroidetes* population and maintains intestinal eubiosis in ethanol-treated mice [34]. The main active ingredient of okra fruit extract is flavonoid. It is broadly acknowledged that flavonoid compounds can modulate intestinal bacteria [44, 45, 47, 90]. Specifically, baicalin, a plant-derived flavonoid, has a variety of biological activities, which inhibits lung injury by manipulating the production of intestinal bacteria and short-chain fatty acids [91], improves metabolic abnormalities and intestinal bacteria, and reduces diet-induced metabolic syndrome [92]. Interestingly, total flavonoids of *Abelmoschus manihot* effectively ameliorate the ulcerative colitis aggravated by chronic stress and improve depressive-like phenotype, disturbed intestinal flora, and intestinal barrier function in chronic stress mice [93]. The aforementioned results supported the intervention effects of OE on intestinal microorganisms in SD rats. Additionally, in all experiments, OE exhibited similar intervention effects to diazepam tablets (positive control), one of the widely prescribed sedative drugs for the treatment of anxiety and sleep disorders [94, 95].

In conclusion, we explored the effects of SD on physiological and behavioral aspects in rats and assessed the changes in stress and inflammatory responses in SD rats. Meanwhile, we concomitantly treated rats with OE during the SD process to innovatively highlight the intervention effects of OE on BGPs and intestinal microorganisms in SD rats. Starting from intestinal microorganisms, the study provided a new entry point for SD treatment. However, this study only conducted the preliminary exploration and lacked in-depth study. Additionally, the percentage of flavonoid compounds in the okra fruit extract and the effects of OE on human health remain to be explored in-depth. Therefore, future studies shall investigate the regulatory mechanism of OE in affecting intestinal microorganisms and BGPs at the molecular level. Moreover, from the perspective of the brain-gut axis, more studies on the relationship between intestinal microorganisms and BGPs are warranted and we shall further estimate the association between intestinal microorganisms and sleep. Furthermore, the flavonoid compounds require to be isolated from okra fruit to investigate their regulatory effects on brain-gut peptides and intestinal microorganisms in SD rats. Exploring the regulatory effects of OE on human intestinal microorganisms and its effects on human health (such as dose and side effects) is also important.

## Figures and Tables

**Table 1:** Primer sequences.

Intestinal bacteria	Forward 5′–3′	Reverse 5′–3′
*Bifidobacteria*	GATGCAACGCGAAGAACCTTACCT	CTTAACCCAACATCTCACGACACGA
*Lactobacilli*	GGGAGGCAGCAGTAGGGAATCTT	GTTAGCCGTGACTTTCTGGTTGGAT
*Clostridium perfringens*	GCGTAGAGATTAGGAAGAACACCAG	TATTCATCGTTTACGGCGTGGACTA
Bacteroidetes	AAAGGGAGCGTAGGTGGACAGTT	TGCCTTCGCAATCGGAGTTCTTC

## Data Availability

All data generated or analyzed during this study are included in the article and can be obtained from the corresponding author upon request.
